# Knowledge about dementia in South Korean nursing students: a cross-sectional survey

**DOI:** 10.1186/s12912-015-0116-4

**Published:** 2015-12-02

**Authors:** Jung Ha Shin, Hyun-Ju Seo, Kye Ha Kim, Kyoung-Hoon Kim, Youngjin Lee

**Affiliations:** Graduate School of Chosun University, Gwangju, South Korea; Department of Nursing, College of Medicine, Chosun University, 309 Pilmum-daero, Dong-gu, Gwangju 61452 South Korea; Department of International Cooperation, Health Insurance Review & Assessment Service, Seoul, South Korea; College of Nursing, Korea University, Seoul, South Korea

**Keywords:** Dementia, Knowledge, Nursing students

## Abstract

**Background:**

The number of individuals with dementia is increasing substantially due to South Korea’s rapidly aging society. Undergraduate nursing students need to have adequate knowledge about dementia to deliver appropriate nursing services. The purpose of this study was to assess the knowledge about dementia among undergraduate nursing students.

**Methods:**

A total of 148 students ranging from freshmen to seniors at a nursing university participated in this study. Data were collected through self-reports using 12-item questionnaires with true/false responses. Knowledge levels about the general characteristics including demographic categories and dementia- related education and training were determined. Factors affecting the score of dementia knowledge were also investigated.

**Results:**

The average score and standard deviation for knowledge about dementia were 10.26 and 1.24 out of 12 points. They had relatively low knowledge about the “prevention and treatment” and “causes” of dementia, with overall correct rate of 78.6 % and 85.4 %, respectively. Higher level of knowledge about dementia was associated with increase in grade level (*p* < 0.001), experience in education on dementia (*p* = 0.01), previous experience in caring for people with dementia during clinical practice (*p* < 0.001), and acquiring information on dementia (*p* = 0.02). Factors that influenced knowledge about dementia included grade level and experience in caring for dementia patients during clinical practice.

**Conclusions:**

This study showed that the level of knowledge about dementia among nursing students was reasonably good. Integrating dementia education and clinical experience into the curricula of undergraduates could improve knowledge about the causes, prevention, and treatment methods for dementia.

## Background

There is a worldwide increase in the number of dementia patients [1]. In South Korea, the prevalence of dementia among older adults over 65 years old was approximately 9.18 % in 2012. And the number of dementia patients is expected to double every 20 years; An estimated 1.27 and 2.71 million will be afflicted in 2030 and in 2050, respectively [2]. Because the prevention, management, and treatment of people with dementia have become an important national issue without being limited to individual anymore, Dementia Control Act was established in February 2012 [3]. As the number of dementia cases is increasing, the chance of nursing university students to directly interact and care for dementia patients is also increasing. A key strategy to develop a workforce capable of providing care for people with dementia is by educating health professionals to improve their understanding about this complex condition [4].

In spite of the increasing burden from dementia, dementia-care related education in nursing universities has been limited to short-term courses or modules instead of detailed coursework in the continuing education system for professional training [5]. A study targeting nurses working at training hospitals has mentioned the need to improve the level of knowledge about dementia because participants only can score 10.8 points out of 16 points [6]. Another study on nurses working in departments related to dementia has reported that nurses need to improve their knowledge in several areas, including effective communication skills with people with dementia and behavioral assessment [7]. In addition, knowledge about caring for dementia patients and practical education of Korean nurses who work at general and long-term care hospitals was ranked the third among nursing requirements [8].

Therefore, nursing university students need to be prepared to enhance nursing care quality and improve the quality of life of patients with dementia [9, 10]. Knowledge about dementia has been assessed in various healthcare professionals, including nursing college students [11], psychology students [12], medical students [13], and health science students majoring in occupational therapy and social welfare [9]. However, no assessment has been performed to understand the knowledge about dementia in nursing undergraduates in all grades in South Korea. Therefore, the objectives of this study were: (1) to investigate the baseline level of knowledge about dementia in undergraduate nursing students, and (2) to compare the knowledge level depending on respondents’ general and educational training characteristics.

## Methods

### Research design

This study had a cross-sectional design to survey the levels of knowledge about dementia through self-reported questionnaires.

### Participants and setting

The target group was full-time undergraduate students from two nursing universities located in a metropolitan city. We selected the two universities due to convenience in sampling to reduce regional differences in data collection. These universities were operating gerontological nursing education using the regular curriculum with the same credits. The target group consisted of 963 full-time students. For ethical considerations, this research was approved by department dean of subject universities. Voluntary participants were recruited by placing posters in subject universities. Additional recruitments were made through describing this study in nursing classes. After receiving permission from the instructor, the primary investigator described the purpose and procedures of this study to nursing classes. Students who agreed to participate then filled out a consent form to confirm their willingness to participate in the research.

We selected a priori power analysis using one-way analysis of variance (ANOVA) to estimate the sample size in a conservative way. Based on previous studies related to this research [14, 15], the effect size was set to large (*d* = 0.40) based on ANOVA (4 groups). Accordingly, the sample size would be large than 76 (large effect). We sampled 154 students considering the possibility of non-participation. A total of 151 students participated voluntarily (response rate: 98.1 %). After excluding three incomplete questionnaires, 148 questionnaires were used for analysis. The 148 participants were considered as an appropriate sample size.

### Measurements

At the time when we performed this research, valid Korean version of measurement instrument was not available to assess the knowledge of dementia among medical or nursing undergraduates. Therefore, the Korean version of Dementia Knowledge Questionnaire developed for lay people and reported as a reliable and valid measure [16] was used in these participants after adding educational and care-related questionnaires [11].

The questionnaire consisted of a total of 12 questions, including three regarding the causes of dementia, four regarding the prevention and treatment of dementia, three regarding the symptom and diagnosis of dementia, and two regarding patient care giving (Table 2). The time for answering each question was set to less than 5 minutes and response was set to be yes or no. The total score ranged from 0 to 12 points, with a high score indicating a high level of knowledge. The Kuder-Richardson 20 score was 0.61, showing moderate internal consistency and reliability. The item-level content validity index (I-CVI) of the questionnaire was examined by five experts with clinical and research experience in the field of psychiatry (*n =* 2) or gerontological nursing (*n =* 3). Based on this questionnaire [16], the mean knowledge scores of participants who indicated that they had been exposed to the principles of dementia at the undergraduate level was 10.3 (SD 1.24), which was significantly (*p* < 0.001) higher than those of lay people with mean score of 9.0 (SD 2.08).

The general characteristics of participants (gender, age, and course progress level) were recorded. Questions were based on educational factors, including interest in dementia, experience in formal dementia education, hours of dementia education (less than 2 h vs. more than 2 h), presence of family members with dementia, personal experience of caring for dementia patients, experience of caring for people with dementia during clinical practicum, exposure to dementia-related information defined as prior experience, obtaining information from mass media, internet, families, relatives, or educational materials, and the type of specific information sources.

### Data collection

The current study protocol was approved by the Institutional Review Board of Chosun University (IRB No: IRB-13-037). After obtaining the approval, the researcher directly visited the targeted educational institution and gained preliminary permission from the dean and advisor after explaining the purpose of the study and the study procedure. The researcher then explained the purpose of the study and distributed questionnaires to students who agreed to participate in this research. Participants were required to sign a consent form and answer the questionnaire. A reward (e.g. a pen of one dollar) was given to students upon completing the survey. Confidentiality/anonymity of the questionnaire was maintained. Data were saved in the researcher’s personal computer with password for security.

### Data analysis

Descriptive statistics were used to understand the general characteristics, factors related to educational training, and the level of dementia knowledge among participants. Scores on dementia knowledge using Kolmogorov-Smirnov test (*p* < 0.001) were not normally distributed. Therefore, we converted the total score to natural logarithms. Two-sample test and one-way ANOVA with Scheffe post hoc analysis were used to compare dementia knowledge levels depending on general characteristics factors related to educational training. Multiple linear regression analysis was used to determine factors affecting the score of dementia knowledge. Variables were selected and entered into multivariate model based on results of univariate analyses (*p* < 0.05). To examine the multi-collinearity of the regression model, variance inflation factor was determined. A variance inflation factor greater than 10 indicated that the model was inadequate [17]. Data analysis was carried out using IBM SPSS Statistics 23.0 (SPSS, Chicago, Illinois, USA). Statistical significance level was set at *p* < 0.05. Marginally significant value was set at 0.05 ≤ *p* < 0.1.

## Results

### Participant characteristics

A total of 148 students were used in this study, including 22 (14.9 %) male students and 126 (85.1 %) female students. Their average age was 21.01 years. The 148 students included 36 (24.3 %) freshmen, 38 (25.7 %) sophomores, 39 (26.4 %) juniors, and 35 seniors (23.6 %). In characteristics related to educational training, 63 (42.6 %) students had dementia education, and 55.6 % of respondents had exposure to dementia-related education with two or fewer hours of dementia education. A total of 84.1 % of participants answered that they had gained a positive attitude toward dementia care after dementia education. In addition, 42.6 % of respondents had experience in caring for persons with dementia. A total of 62 (41.9 %) participants said that they had experience during clinical training session of their program. In addition, 75.0 % of respondents had gained information on dementia from education materials from broadcasting (43.2 %), the internet (27.0 %), and family members or relatives (16.2 %) (Table 1).

**Table 1:** Socio-demographic and dementia related characteristics

Characteristic (*N =* 148)	Number	Percent
Gender		
Female	126	85.1
Grade level		
Freshman	36	24.3
Sophomore	38	25.7
Junior	39	26.4
Senior	35	23.6
Experience of dementia education		
Yes	63	42.6
Average time of Experienced education (*n =* 63)		
Less than 2 h	35	55.6
More than 2 h	28	44.4
Family member with dementia		
Yes	24	16.2
Experience in caring for persons with dementia		
Yes	63	42.6
Cared for people with dementia during clinical placement		
Yes	62	41.9
Exposure to information on dementia		
Yes	111	75.0
Information sources^a^ (*n =* 111)		
Broadcasting	64	43.2
Educational resources	64	43.2
Internet	40	27.0
Family/relatives	24	16.2
Newspaper	21	14.2
Magazine	2	1.4
Others	10	6.8

^a^Multiple responses possible

### Knowledge level about dementia

The average knowledge level about dementia was 10.26 ± 1.24 points out of a total of 16 points, equivalent to a score of 85.5 on a scale of 100. Questions addressing whether “regular exercise reduces the risk of dementia,” and whether “because a dementia patient has no ability to judge, the patient does not need any explanation as to how he or she is cared for” received the highest score, with 95.9 % of respondents answered correct. The lowest correct answer rate was 37.2 % for the question on whether “some types of dementia can be cured completely”. As for the correct answer rates according to knowledge area of dementia, the average correct answer rates in the care giving area, symptoms and diagnosis area, causes of dementia area, and prevention and treatment area were 94.6 %, 89.0 %, 85.4 %, and 78.6 %, respectively (Table 2).

**Table 2:** Percentage of correct answers per question on knowledge about dementia

Category	Questionnaire item	True/false	Item correct (%)	Overall correct (%)
Causes	Everyone develops dementia when he or she gets old.	F	91.9	85.4
	Alzheimer's disease is the most common cause of dementia.	T	78.4	
	Stroke may lead to dementia.	T	85.8	
Prevention and treatment	There is no way to prevent dementia.	F	95.3	78.6
	Some types of dementia can be cured completely.	T	37.2	
	Drugs are useful to treat dementia.	T	85.8	
	Regular exercise reduces the risk of dementia.	T	95.9	
Symptom and diagnosis	If a person remembers well what happened a long time ago, he or she does not have dementia.	F	84.5	89.0
	If a person develops dementia, he or she may experience change in his or her personality.	T	93.9	
	Dementia can be determined only when strange behaviors appear.	F	88.5	
Caregiving	If a person develops dementia, it is impossible for him or her to live with his or her family.	F	93.2	94.6
	Because a dementia patient has no ability to judge, the patient does not need any explanation as to how he or she is cared for.	F	95.9	

### Differences in knowledge level according to general characteristics of participants

The knowledge level depending on participants’ general characteristics and relevant educational training showed that the more advanced courses that the participants were in, the higher their knowledge level (*p* < 0.001). Respondents with educational training experience presented higher knowledge levels (*p* = 0.01) than others. In addition, respondents with patient caring experiences during clinical placement had a significantly (*p* < 0.001) higher knowledge level than those without experience in clinical practice. Respondents with exposure to information on dementia presented a higher (*p* = 0.02) average knowledge level (Table 3).

**Table 3:** Differences in dementia knowledge according to general characteristics

Variables		*n*	*t or F*	*p* ^*^	Scheffé
Gender	Male	22	−0.14	0.88	
	Female	126			
Grade level	First^a^	36	7.04	<0.001^*^	a,b < c,d
	Second^b^	38			
	Third^c^	39			
	Fourth^d^	35			
Dementia education	Yes	63	2.60	0.01^*^	
	No	85			
Mean hours of education	Less than 2 h	41	0.90	0.36	
	More than 2 h	22			
Family member with dementia	Yes	24	−1.27	0.21	
	No	124			
Experience in caring for persons with dementia	Yes	63	0.58	0.56	
	No	85			
Cared for people with dementia during clinical placement	Yes	62	−3.79	<0.001^*^	
	No	86			
Exposure to information of dementia	Yes	111	−2.25	0.02^*^	
	No	37			

^*^ *p* < 0.05

Difference was statistically significant between a, b and c, d by Scheffe's post-hoc comparison

### Factors affecting the score of dementia knowledge

Our results revealed that factors associated with score of dementia knowledge included grade level (junior vs. freshman: coefficient = 0.06, *p* = 0.02; senior vs. freshman: coefficient = 0.06, *p* = 0.03) and experience with caring for dementia patients during clinical placement (ever vs. never: coefficient = 0.031, *p* = 0.08). The variance inflation factor in this regression model was less than 2.815. The adjusted R^2^ value was 11.1 % (Table 4).

**Table 4:** Factors affecting the score of dementia knowledge based on multiple linear regression analysis

Variables	*B*	SE	beta	t	*p*
Grade level					
First	referent				
Second	0.01	0.02	0.06	0.65	0.51
Third	0.06	0.02	0.29	2.25	0.02^*^
Fourth	0.06	0.02	0.27	2.27	0.03^*^
Dementia education	−0.01	0.02	−0.06	−0.53	0.59
Cared for people with dementia during clinical placement	0.03	0.02	0.16	1.75	0.08^**^
Exposure to information of dementia	0.01	0.02	0.05	0.59	0.55

R^2^ = 0.147, Adjusted R^2^ = 0.111, F = 4.065, *P* = 0.001

^*^ *p* <0.05 and **p <0.1

## Discussion

The primary objective of this study was to investigate the knowledge level about dementia among nursing students. Their average knowledge level was found to be favorable, with a score of 10.26 out of a possible 12 points, which was equivalent to 86 points on a scale of 100 points. Their knowledge level in the causes of dementia and the prevention and treatment areas needs to be improved compared to knowledge in care giving and the symptoms and diagnosis of dementia. Several international studies have reported relatively lower levels of knowledge regarding dementia [9, 11, 12]. The level of knowledge about dementia in nursing college students with a three-year course has been reported to be 64.5 points out of 100 points using Alzheimer’s Disease Knowledge Scale (ADKS) [11]. Using Alzheimer’s disease Knowledge Test (ADKT), it has been reported that college students majoring in health science and social welfare have a mean score 37.0 points out of 100 points [9], and those majoring in psychology have a mean score of 66.0 points [13]. In this study, the knowledge levels of students measured by the Korean version of Dementia Knowledge Questionnaire were relatively higher than those reported in previous studies. This could be due to differences in target populations and the measurement tools. The measurement tool used in this study was developed for the lay public [16]. In contrast, ADKS was developed for university students, health care professionals, and experienced clinical nurses [18]. ADKT was developed for professionals and other health personnel involved in the care of Alzheimer’s patients [13]. In addition, the measurement instrument used in this study was relatively easy in terms of indexing difficulty, which might have resulted in higher score of dementia knowledge compared to those reported in previous studies.

Our second objective was to examine the mean differences in dementia knowledge by general characteristics and determine the factors related to the educational training. The factor that was the most influential for dementia knowledge was grade level. Experience in caring for people with dementia during clinical placement was marginally significant. Therefore, junior and senior students had more chance to receive dementia-care related education and clinical practices. Our results were consistent with the findings of previous studies targeting nursing undergraduates [9, 11] and medical school students [19]. In addition, participants who had received dementia education and clinical placement showed significantly higher dementia knowledge levels than others, corresponding to a previous report that dementia knowledge levels in college students were significantly increased after dementia education programs [12, 14]. Our results was particularly similar to a previous study on the knowledge of Alzheimer’s disease among health care staff [20]. Therefore, it is necessary to provide dementia-care-related educational curricula and clinical training in order to improve the knowledge and skills required for nursing personnel who have direct contact with patients/public across health and social care areas [21].

The level of dementia knowledge in those who had exposure to dementia information was significantly higher than that in others. The main sources of information depending on grade level of students were mass media (for 70 % of freshmen and 80 % of sophomores) and educational materials (for 74 % of juniors and 79 % of seniors). However, because a majority (54.7 %) of respondents answered that they had two or fewer hours of dementia education, it was difficult to determine if there was a regular education course regarding dementia. In order to estimate the status of dementia care related education in South Korea, we reviewed a nation-wide survey that investigated the level of gerontological nursing education in bachelor of science nursing programs offered by Korean Universities. Thirty-four (66.6 %) of 51 respondents at four-year nursing universities reported that gerontological nursing was elective courses, while 54.9 % of participants reported that credits for gerontological nursing were “very insufficient” or “insufficient” [22].

As the prevalence of dementia is expected to continuously increase in the rapid aging society, it is important to raise knowledge level about dementia and train nurses and caregivers so that they can provide appropriate services to dementia patients. Therefore, nursing schools need to operate education programs to provide dementia-care-related education and clinical practicum on dementia, including the symptoms and difficulties of dementia, risk factors, screening and diagnosis, disease progress, influences on life stages of individuals with dementia, impact on family and individual, communications, patient care, treatment and management of the illness, environmental aspects, and equality [21].

Regarding the dementia knowledge levels, students who had family members with dementia showed significantly higher levels than those who did not have family members with dementia. This was in agreement with a previous report that students who lived with older family members with dementia had a higher knowledge level [23]. This could be explained by a previous finding that the pursuit of dementia-related interests or dementia knowledge was increased among those who had family members with dementia [24].

The present study was vulnerable to selection bias because it sampled participants from two nursing universities in the same metropolitan city. Another limitation of this study was that we did not find any plausible tool verified in the Korean context to measure the dementia knowledge level of nursing students. In 2015, a study published the psychometric properties using the Korean version of the Alzheimer’s Disease Knowledge Scale (ADKS-K) and determined its applicability for Korean adults [25]. Therefore, we evaluated our study participants using the revised tool aided by education and care related items. Further studies are needed to evaluate the dementia knowledge level of nursing students using methodologies validated in the Korean context. Nevertheless, to the best of our knowledge, this study provided important information. It was the first study that examined the baseline dementia knowledge level of Korean nursing students. In addition, we determined the factors related to education training affecting dementia knowledge.

## Conclusions

The study revealed that the knowledge level about dementia among undergraduate nursing students was good, with a mean point of 10.26 out of a possible 12 points. The knowledge level was significantly higher for senior students and those who had experience with dementia education, obtained dementia information, had experience caring for dementia patients during clinical practice practicum, and had family members with dementia. Factors that could influence the knowledge about dementia included grade level of students and experience in caring for dementia patients during clinical placement. These findings suggest that nursing schools need to introduce and operate dementia care related education programs to improve the dementia knowledge in undergraduate nursing students.
